# Benefits of protected areas for nonbreeding waterbirds adjusting their distributions under climate warming

**DOI:** 10.1111/cobi.13648

**Published:** 2021-01-21

**Authors:** Elie Gaget, Diego Pavón‐Jordán, Alison Johnston, Aleksi Lehikoinen, Wesley M. Hochachka, Brett K. Sandercock, Alaaeldin Soultan, Hichem Azafzaf, Nadjiba Bendjedda, Taulant Bino, Luka Božič, Preben Clausen, Mohamed Dakki, Koen Devos, Cristi Domsa, Vitor Encarnação, Kiraz Erciyas‐Yavuz, Sándor Faragó, Teresa Frost, Clemence Gaudard, Lívia Gosztonyi, Fredrik Haas, Menno Hornman, Tom Langendoen, Christina Ieronymidou, Vasiliy A. Kostyushin, Lesley J. Lewis, Svein‐Håkon Lorentsen, Leho Luigujõe, Włodzimierz Meissner, Tibor Mikuska, Blas Molina, Zuzana Musilová, Viktor Natykanets, Jean‐Yves Paquet, Nicky Petkov, Danae Portolou, Jozef Ridzoň, Samir Sayoud, Marko Šćiban, Laimonas Sniauksta, Antra Stīpniece, Nicolas Strebel, Norbert Teufelbauer, Goran Topić, Danka Uzunova, Andrej Vizi, Johannes Wahl, Marco Zenatello, Jon E. Brommer

**Affiliations:** ^1^ Department of Biology University of Turku Turku 20500 Finland; ^2^ Department of Terrestrial Ecology Norwegian Institute for Nature Research (NINA) P.O. Box 5685 Sluppen Trondheim N‐7485 Norway; ^3^ Cornell Lab of Ornithology Cornell University Ithaca NY 14850 U.S.A.; ^4^ Conservation Science Group, Department of Zoology University of Cambridge Cambridge CB2 3QZ U.K.; ^5^ The Finnish Museum of Natural History University of Helsinki P.O. Box 17 Helsinki 00100 Finland; ^6^ Department of Ecology Swedish University of Agricultural Sciences Uppsala 750 07 Sweden; ^7^ Association "Les Amis des Oiseaux" (AAO/BirdLife en Tunisie) 14, Rue Ibn El Heni, 2ème étage ‐ Bureau N° 4 Ariana 2080 Tunisia; ^8^ Direction générale des Forêts Ben Aknoun Alger 16000 Algérie; ^9^ Albaninan Ornithological Society Bulevardi "Gjergj Fishta" Kulla nr.2, kati 4, hyrja 18 Tirana 1001 Albania; ^10^ Društvo za opazovanje in proučevanje ptic Slovenije (DOPPS) Tržaška cesta 2 Ljubljana SI‐1000 Slovenia; ^11^ Department of Bioscience Aarhus University Rønde 8200 Denmark; ^12^ Scientific Institute Mohammed V University of Rabat Av. Ibn Battota Rabat‐Agdal 10106 Morocco; ^13^ Research Institute for Nature and Forest Brussel 1070 Belgium; ^14^ Romanian Ornithological Society Bd. Hristo Botev, nr.3, ap. 6, Sector 3 Bucureşti 030231 Romania; ^15^ Instituto da Conservação da Natureza e das Florestas, IP (ICNF) Centro de Estudos de Migrações e Proteção de Aves (CEMPA) Lisboa 1050‐191 Portugal; ^16^ Ornithological Research Center Ondokuz Mayis University Samsun 55139 Turkey; ^17^ Institute of Wildlife Management and Vertebrate Zoology University of Sopron Bajcsy‐Zsilinszky u. 4 Sopron H‐9400 Hungary; ^18^ British Trust for Ornithology Thetford IP24 2PU U.K.; ^19^ LPO‐BirdLife France Fonderies Royales Rochefort Cedex 17300 France; ^20^ Department of Biology Lund University Lund 223 62 Sweden; ^21^ Sovon Dutch Centre for Field Ornithology Nijmegen 6525 ED The Netherlands; ^22^ Wetlands International Ede 6717 LZ Ede The Netherlands; ^23^ BirdLife Cyprus P.O Box 12026 Nicosia 2340 Cyprus; ^24^ Monitoring and Animal Conservation Department, Schmalgausen Institute of Zoology NAS of Ukraine vul. B. Khmelnytskogo, 15 Kyiv 01030 Ukraine; ^25^ I‐WeBS Office BirdWatch Ireland Wicklow A63 RW83 Ireland; ^26^ Department of Zoology Estonian University of Life Sciences Tartu 51006 Estonia; ^27^ Department of Vertebrate Ecology and Zoology, Faculty of Biology University of Gdańsk Wita Stwosza 59 Gdańsk 80–308 Poland; ^28^ Croatian Society for Bird and Nature Protection Zagreb 1000 Croatia; ^29^ Sociedad Española de Ornitología (SEO/BirdLife) Madrid 28053 Spain; ^30^ Faculty of Environmental Sciences Czech University of Life Sciences Praha Suchdol 129 Kamýcká CZ‐165 21 Czechia; ^31^ National Academy of Science of Belarus Independence Avenue 66 Minsk 220072 Republic of Belarus; ^32^ Département Études Aves‐Natagora Namur 5000 Belgium; ^33^ Bulgarian Society for the Protection of Birds PO Box 50 Sofia BG‐1111 Bulgaria; ^34^ Hellenic Ornithological Society Themistokleous str. 80 Athens 10681 Greece; ^35^ SOS/BirdLife Slovakia Bratislava 821 08 Slovakia; ^36^ Bird Protection and Study Society of Serbia Vladike Ćirića 24/19, 21000 Novi Sad, Srbija Makedonska 4 Beograd 11000 Srbija; ^37^ Lithuanian Ornithological Society Naugarduko 47‐3 Vilnius LT‐03208 Lithuania; ^38^ Institute of Biology University of Latvia Salaspils LV‐2169 Latvia; ^39^ Swiss Ornithological Institute Sempach CH‐6204 Switzerland; ^40^ BirdLife Österreich Museumsplatz 1/10/8 Vienna 1070 Austria; ^41^ Nase Ptice Ornithological Society Sarajevo BA–71000 Bosnia and Herzegovina; ^42^ Macedonian Ecological Society Boris Trajkovski st. 7 No. 9A Skopje 1000 Macedonia; ^43^ Natural History Museum of Montenegro Trg Vojvode Bećir‐bega Osmanagića 16 Podgorica 81000 Montenegro; ^44^ Dachverband Deutscher Avifaunisten e.V. (DDA) Federation of German Avifaunists Münster 48157 Germany; ^45^ Istituto Superiore per la Protezione e la Ricerca Ambientale (ISPRA) Ozzano dell'Emilia 40064 Italy

**Keywords:** colonization, community adjustment, community temperature index, extinction, range shift, wetlands, ajuste comunitario, cambio de distribución, colonización, extinción, humedales, índice de temperatura comunitaria, 定殖, 群落调整, 群落温度指数, 灭绝, 范围变化, 湿地

## Abstract

Climate warming is driving changes in species distributions and community composition. Many species have a so‐called climatic debt, that is, shifts in range lag behind shifts in temperature isoclines. Inside protected areas (PAs), community changes in response to climate warming can be facilitated by greater colonization rates by warm‐dwelling species, but also mitigated by lowering extirpation rates of cold‐dwelling species. An evaluation of the relative importance of colonization‐extirpation processes is important to inform conservation strategies that aim for both climate debt reduction and species conservation. We assessed the colonization‐extirpation dynamics involved in community changes in response to climate inside and outside PAs. To do so, we used 25 years of occurrence data of nonbreeding waterbirds in the western Palearctic (97 species, 7071 sites, 39 countries, 1993–2017). We used a community temperature index (CTI) framework based on species thermal affinities to investigate species turnover induced by temperature increase. We determined whether thermal community adjustment was associated with colonization by warm‐dwelling species or extirpation of cold‐dwelling species by modeling change in standard deviation of the CTI (CTI_SD_). Using linear mixed‐effects models, we investigated whether communities in PAs had lower climatic debt and different patterns of community change than communities outside PAs. For CTI and CTI_SD_ combined, communities inside PAs had more species, higher colonization, lower extirpation, and lower climatic debt (16%) than communities outside PAs. Thus, our results suggest that PAs facilitate 2 independent processes that shape community dynamics and maintain biodiversity. The community adjustment was, however, not sufficiently fast to keep pace with the large temperature increases in the central and northeastern western Palearctic. Our results underline the potential of combining CTI and CTI_SD_ metrics to improve understanding of the colonization‐extirpation patterns driven by climate warming.

## Abstract 2

*Article impact statement*: Protected areas are needed to facilitate waterbird distribution change in response to climate warming in the western Palearctic.

## Abstract (es)

Beneficios de las Áreas Protegidas para las Aves Acuáticas No Reproductoras que Están Ajustando su Distribución Debido al Calentamiento Climático

**Resumen**

El calentamiento climático está generando cambios en la distribución y en la composición comunitaria de las especies. Muchas de ellas tienen una deuda climática, es decir, los cambios en la distribución se atrasan con respecto a los cambios en las isoclinas térmicas. Dentro de las áreas protegidas (APs), los cambios comunitarios como respuesta al calentamiento climático pueden facilitarse mediante tasas mayores de colonización por especies de climas cálidos, pero también pueden mitigarse al reducir las tasas de extirpación de las especies de climas fríos. Se requiere una evaluación de la importancia relativa de los procesos de colonización‐extirpación para orientar las estrategias de conservación que buscan la reducción de la deuda climática y la conservación de las especies. Analizamos las dinámicas de colonización‐extirpación que participan en los cambios comunitarios como respuesta al clima dentro y fuera de las APs. Para realizar lo anterior, usamos datos tomados durante 25 años de la presencia de aves acuáticas no reproductoras en el Paleártico occidental (97 especies, 7,071 sitios, 39 países, 1993–2017). Usamos un marco de trabajo del índice de temperatura comunitaria (ITC) basado en las afinidades térmicas de las especies para así investigar la rotación de especies inducida por el incremento en la temperatura. Determinamos si el ajuste térmico en la comunidad estuvo asociado con la colonización por especies de climas cálidos o con la extirpación de especies de climas fríos al modelar el cambio mediante una desviación estándar del ITC (ITC_DS_). Con los modelos lineales de efectos mixtos investigamos si las comunidades dentro de las APs tenían una deuda climática más baja y patrones diferentes de cambio comunitario que las comunidades localizadas fuera de las APs. Con la combinación del ITC y deL ITC_DS_, las comunidades dentro de las APs tuvieron más especies, una mayor colonización, una menor extirpación y una deuda climática más baja (16%) que las comunidades fuera de las APs. Por lo tanto, nuestros resultados sugieren que las APs facilitan dos procesos independientes que moldean las dinámicas comunitarias y mantienen la biodiversidad. Sin embargo, el ajuste comunitario no fue lo suficientemente rápido para mantener el paso de los grandes incrementos en la temperatura de las regiones central y noreste del Paleártico occidental. Nuestros resultados resaltan el potencial que tiene la combinación de las medidas del ITC y del ICT_DS_ para mejorar el entendimiento de los patrones de colonización‐extirpación causados por el calentamiento climático.

## 【摘要】

气候变暖正在引起物种分布和群落组成的变化。许多物种都背负着所谓的 “气候债务”, 即分布范围的变化滞后于温度的变化。气候变暖背景下, 保护地中适应温暖环境的物种较高的定殖率可以促进群落变化, 适应寒冷环境的物种灭绝率下降也可以减缓影响。评估定殖–灭绝过程的相对重要性, 对于制定旨在减少气候债和促进物种保护的保护战略而言十分重要。本研究利用西古北界非繁殖水鸟 25 年的出现记录 (97 个物种, 7071 个位点, 39 个国家, 1993–2017 年) , 评估了保护地内外鸟类群落响应气候变化过程中的定殖–灭绝动态。我们使用基于物种对温度亲和性的群落温度指数框架探究了温度升高引起的物种更替。通过模拟群落温度指数标准偏差的变化, 我们确定了群落对温度的调整是否与适应温暖环境的物种的定殖或适应寒冷环境的物种的灭绝有关。接下来, 我们利用线性混合效应模型分析了保护地内与保护地外的群落相比是否具有较低的气候债务和不同的群落变化格局。结合群落温度指数与其标准偏差, 我们发现保护地内的群落比保护地外的具有更多的物种、更高的定殖率、更低的灭绝率和更低的气候债务 (16%) 。因此, 我们的结果表明, 保护地促进了群落动态形成和生物多样性维持这两个独立过程。然而, 群落调整的速度不足以跟上古北界中部和东北部地区气温的大幅上升。我们的结果强调了群落温度指数和群落温度指数标准偏差这两个指标相结合, 有潜力提高对气候变暖导致的定殖–灭绝格局的理解。**【翻译: 胡怡思; 审校: 聂永刚】**

## Introduction

Global warming is one of the major causes of biological changes among the growing number and variety of anthropogenic pressures on the natural world (Monastersky 2014). One of the clearest biological signals of environmental change has been global species distribution shifts toward the poles (Parmesan & Yohe 2003; Chen et al. 2011), which are driven by colonization at the leading distribution edge and extirpation at the trailing edge (Thomas & Lennon 1999). However, distribution changes have mostly been insufficient to track the thermal isocline shifts that lead to climatic debt in species distributions (Chen et al. 2011; Devictor et al. 2012). Furthermore, the pressures from climate change may be exacerbated by other environmental factors that can interact with colonization and extirpation processes (Hill et al. 2001; Brook et al. 2008), such as habitat fragmentation (Hill et al. 2001) or land‐use change (Auffret & Thomas 2019; Gaget et al. 2020). However, protected areas (PAs) may mediate these other pressures and facilitate species’ responses to climate change (Thomas et al. 2012).

Protected areas are expected to facilitate species distribution shifts in response to climate warming by reducing anthropogenic pressures on ecosystems (Thomas et al. 2012). Defined as areas set aside and managed for the purpose of conservation, PAs (UNEP‐WCMC, IUCN and NGS 2021) are one of the most efficient ways to protect ecosystems of high biological importance (Godet & Devictor 2018). At the leading edge of species distributions, colonization may be more likely to occur in PAs (Hiley et al. 2013; Gillingham et al. 2015; Lehikoinen et al. 2019; Peach et al. 2019), particularly with large PA surface (Gaüzère et al. 2016), and to promote range expansion (Thomas et al. 2012; Pavón‐Jordán et al. 2015). Conversely, species extirpation at the trailing edge can be reduced in PAs (Gillingham et al. 2015; Lehikoinen et al. 2019; Peach et al. 2019). In view of these contrasting patterns, it is important to evaluate in a comprehensive framework the effects of PAs on changes in species distribution throughout the overall community of species.

**Figure 1:**
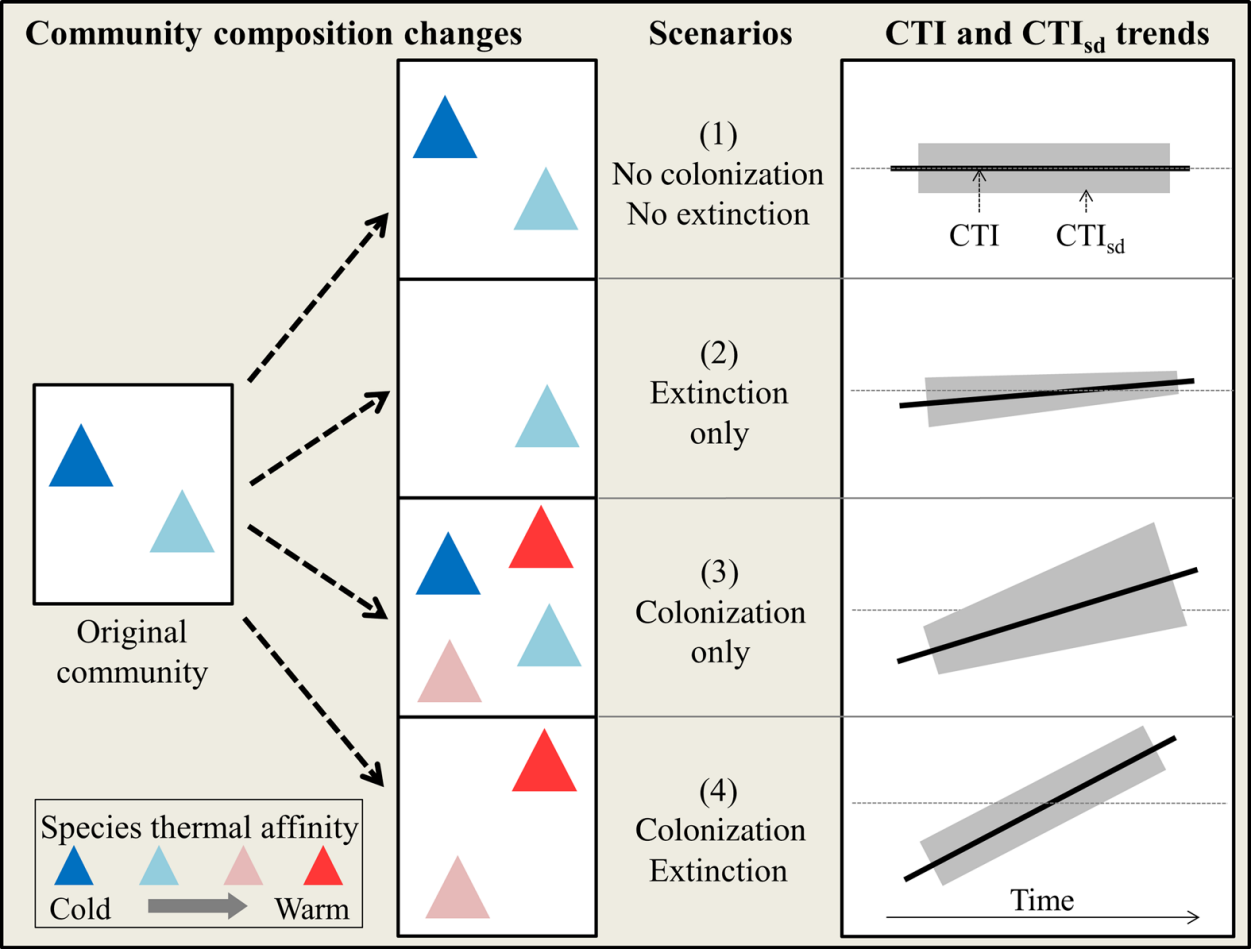
Models of the 4 theoretical scenarios of species colonization or extirpation relative to species thermal affinities (triangles, species). Community changes in response to climate warming are revealed by trends of community temperature index (CTI) (i.e., thermal average) and CTI standard deviation (CTI_SD_) over time (Gaüzère et al. 2019). The CTI slopes depend on both rate of colonization or extirpation and on the species temperature index values.

Temperature‐driven shifts in species distributions will reshuffle community structure; warm‐dwelling species will colonize and cold‐dwelling species will be extirpated (Devictor et al. 2008). Figure 1 illustrates how the community adjustment to climate warming can be assessed with the intuitive community temperature index (hereafter, CTI), by measuring changes in community composition as a function of all species’ thermal affinities (Devictor et al. 2008). In addition to the average community response measured with the CTI, the variance of the response provides a complementary indicator for conservation assessments that can be used to investigate species’ colonization‐extirpation processes relative to species’ thermal affinities (Fig. 1) (Gaüzère et al. 2019, Supporting Information). Indeed, a community adjustment to climate warming may involve mainly extirpation of cold‐dwelling species (Fig. 1, scenario 2) or colonization by warm‐dwelling species (Fig. 1, scenario 3), which have different conservation implications. In addition, the CTI allows one to identify how local conditions, such as site protection, influence community adjustment to warming (Gaüzère et al. 2016; Santangeli & Lehikoinen 2017) and quantify any delay in tracking climate warming, namely the climatic debt (Devictor et al. 2012).

We investigated the community adjustment of nonbreeding waterbirds to climate warming throughout the western Palearctic over 25 years and tested whether the long‐term patterns of community change differed inside and outside PAs. The survey region, extending from the Mediterranean biodiversity hotspot to the fast‐warming Arctic, faces substantial anthropogenic pressures (IPBES 2018a; IPCC ; 2018b). Despite great conservation efforts, there has been considerable loss and degradation of wetlands in this region (Dixon et al. 2016) and many waterbird populations have been declining for decades (Gardner & Davidson 2011). For these reasons, waterbirds have been targeted with a large‐scale monitoring program, the International Waterbird Census (IWC) (Delany 2010). Data collection for the IWC is particularly intensive and extensive in western Palearctic countries, both inside and outside PAs (Delany 2010), and provides unique data to investigate the effectiveness of conservation strategies at continental scale (Pavón‐Jordán et al. 2015; Amano et al. 2018; 2020). Indeed, numerous studies have identified change in the nonbreeding distributions of waterbirds in response to climate warming in the western Palearctic (Maclean et al. 2008; Lehikoinen et al. 2013; Pavón‐Jordán et al. 2019) that are related to conservation measures (Johnston et al. 2013; Pavón‐Jordán et al. 2015; Gaget et al. 2018). However, assessments at the community level inside and outside PAs are still lacking. We predicted that in response to climate warming, warm‐dwelling waterbirds will expand their distribution by colonizing more PAs and cold‐dwelling species may be more resilient in PAs (Fig. 1 scenario 3) independent of the year that PAs received their conservation designation because PAs usually contain high‐quality habitat even prior to designation (Lawson et al. 2014).

We analyzed an extensive data set on waterbird occurrence (97 species) across 39 countries (7071 sites). We examined community dynamics within the CTI framework and the related community thermal standard deviation (CTI_SD_) (Fig. 1) to address 3 objectives: to determine whether the community adjustment to climate warming was higher and the climatic debt lower inside PAs; to identify whether in PAs there was more colonization by warm‐dwelling species and fewer extirpations of cold‐dwelling species; and to investigate whether the community adjustment to climate warming was positively related to local PA coverage. A community response to climate change is assumed to be better when the CTI trend is more positive.

## Methods

### Study Area and Waterbird Monitoring

We used IWC data from almost all of the western Palearctic (39 countries with sufficient data according to criteria below [Supporting Information]) from 1993 to 2017. The IWC monitors nonbreeding waterbirds (i.e., overwintering populations) with a single count each year in January by ornithologists, either professionals or citizen scientists. The count is coordinated by Wetlands International (www.wetlands.org) (see Delany [2010] for the protocol). The IWC monitors all wetland types, including both protected and unprotected sites, and one of the main goals is the assessment of the effectiveness of waterbird conservation policies. To ensure a long‐term survey of community changes, we used information from the 7071 sites that had at least 5 counts, 1 count in the 1990s, 2000s, and 2010s (mean 16.6 counts per site [SD 5.6]), and at least 2 species per count (*n* = 117,325 counting events [Supporting Information]). The data used for the analyses included 97 species of nonvagrant waterbirds that overwinter in the western Palearctic (Supporting Information) and are listed in the African‐Eurasian Migratory Waterbird Agreement (http://www.unep-aewa.org).

### Protected Areas and Temperature Data

Site protection is reported for 3374 sites from the World Database on Protected Areas (UNEP‐WCMC, IUCN and NGS 2021), the Natura 2000 database, and the Common Database on Designated Areas (www.eea.Europa.eu) (Supporting Information). We included all levels of International Union for Conservation of Nature (IUCN) PA management category (I–VI), following the definition given by UNEP‐WCMC, IUCN and NGS (2021). A site was considered a PA when its coordinates fell within the polygon of a PA designated before 2017, meaning that we investigated the effect of the area where the PA was established rather than the effect of PA designation. If polygon data were absent (12% of the cases), a circular area was delineated based on the PA size reported in the World Database on Protected Areas (100% concordance of site‐protection status was found by delineating a circular area on the subset of PAs with polygons). The sites inside (*n* = 3374) and outside (*n* = 3697) PAs had a similar number of counts and spatial distribution (Supporting Information).

We compiled temperature data for our study sites from the HadCRUT4 data set (Morice et al. 2012), which has a spatial resolution of 0.5°. Yearly winter temperatures, likely influencing waterbird overwintering location, were computed each winter as the average of the mean monthly temperatures for November, December, and January.

### Community Temperature Indices

Winter species temperature indices (STIs) were computed as the species thermal affinity across each species’ nonbreeding distribution following Gaget et al. (2018) (adapted for nonbreeding waterbirds from Devictor et al. [2008]). The winter STI is the long‐term average temperature in January (WorldClim database, 1950–2000, http://worldclim.org/) experienced by the species across the nonbreeding (overwintering) distribution during the monitoring period (extracted from BirdLife International & Handbook of the Birds of the World [2018]). Subspecies with distributions in Sub‐Saharan Africa were removed to avoid possible overestimation of the winter temperatures experienced by our study populations (Supporting Information).

The CTI and CTI standard deviation (CTI_SD_) were computed following Devictor et al. (2008) and Gaüzère et al. (2019) on species occurrence (presence–absence). The CTI is the average STI of the species present in the community per count event (Supporting Information). The CTI_SD_ is the standard deviation of the species STI present in the community per count event and quantifies STI heterogeneity in the community. Thus, the CTI increases over the years when a community includes more warm‐dwelling species or fewer cold‐dwelling species. In contrast, the CTI_SD_ increases over the years when the thermal affinities of the community become more heterogeneous (Fig. 1). Occurrence data were used instead of abundance data to make it easier to interpret the processes of colonization and extirpation (Supporting Information).

### Protected Areas, CTI, CTI_SD_, and Climatic Debt

Temporal changes in temperature, CTI, and CTI_SD_ that depended on PA status were assessed with generalized linear mixed effects models (GLMM) (Gaussian error distribution). The explanatory terms were year (continuous variable from 1993 to 2017), site protection status (inside or outside), and the interaction of year × protection status. Site identity was added as a random effect on the intercept in the CTI and CTI_SD_ models. Spatial autocorrelation was taken into account by including the site geographical coordinates as an exponential spatial correlation structure in the model (Gaget et al. 2018). The temperature was not included as a dependent variable in the models so that we could measure the climatic debt as defined by Devictor et al. (2008). The linear model was

(1) $$T_{i}{,}_{j}∼\;\mu +year_{i}\times PA_{j}+site_{j}+\varepsilon_{i}{,}_{j},$$

where *T_ij_* is the temperature, CTI, or CTI_SD_, in year *i* at site *j*, *μ* is the intercept, PA is the site protection status of site *j*, site is the random intercept per site (follows a Gaussian distribution; mean of zero and variance σ²), and ε is the residual variance for each observation under a Gaussian distribution and an exponential spatial correlation. To visually assess whether it was appropriate to model interannual changes as a linear effect, we generated and plotted mean annual values (95% CI) by using the same model, but changing year to a categorical variable. We conducted complementary analyses to assess the robustness of the results to species’ identity and abundance with a resampling approach. Resampling followed Devictor et al. (2012), in which the CTI and CTI_SD_ trends were estimated after the random removal of 20% of the species (1000 iterations) based on occurrence and abundance data (Supporting Information).

We looked for evidence of climatic debt accumulated by the waterbird communities by assessing the difference between the linear trends of temperature and CTI, following Devictor et al. (2008). First, we investigated the latitudinal gradients in temperature and CTI with a linear model with latitude as a fixed effect. The latitudinal gradient was converted to kilometers by multiplying units in degrees by 111.128 (i.e., the average number of kilometers per 1 decimal degree over the whole study area). Then the temporal temperature change (degrees Celsius per year) was converted to a velocity of temperature change (kilometers per year) by using the latitudinal temperature gradient (degrees Celsius per kilometer) from south to north of the study area. The same steps were taken with the CTI. Last, the climatic debt was obtained by subtracting the velocity of the CTI change from the velocity of the temperature change over the study period.

In addition, we assessed the temporal trend of cold‐ and warm‐dwelling species inside versus outside PAs to illustrate the absolute changes of thermal‐dwelling composition in the communities. We used 2 simplified thermal‐dwelling categories to classify species as cold dwelling or warm dwelling based on their STI in relation to the CTI of each individual site: cooler or warmer STI than the mean CTI across the site's whole time series, respectively. Then, the number of cold‐ and warm‐dwelling species was summed per survey. The temporal changes in number of cold‐ and warm‐dwelling species were assessed using in a GLMM (Poisson error distribution) with fixed effects of year, thermal‐dwelling category (cold or warm), site PA status (inside or outside), and their 3‐way interaction. Site identity was added as a random factor. Spatial autocorrelation was taken into account by including the site geographical coordinates as an exponential spatial correlation structure in the model. The GLMM was

$$N_{i,j,k}∼\;\mu +year_{i}\times PA_{j}\times D_{k}+site_{j}+\varepsilon_{i,j,k},$$

where *N_i,j,k_* is the number of species summed per survey in year *i* at site *j* for the thermal‐dwelling category *k*, *μ* is the intercept, PA is the site protection status of site *j*, *D* is the thermal‐dwelling category (cold or warm), site is the random intercept per site that follows a Gaussian distribution (mean of 0 and variance σ²), and *ε* is the residual variance for each observation under a Gaussian distribution and an exponential spatial correlation.

### Community Changes and Proportion of PA Surface

We investigated whether the CTI, climatic debt, and CTI_SD_ trends were correlated with the proportion of PA surface with a moving‐window approach. First, we performed one GLMM per cell (1032 cells of 5° × 5° resolution [approximately 500 × 500 km] shifting by one latitudinal or longitudinal degree between the closest cells) per response variable (temperature, CTI, and CTI_SD_) to investigate changes in cells over time. We used site identity as a random effect and included the site geographical coordinates as an exponential spatial correlation structure. Second, we investigated the relationship between the proportion of PA surface per cell and the CTI spatial shift, CTI_SD_, or climatic debt trends (estimated from the above models). The proportion of PA surface was assessed per cell as the sum of the studied site inland PA surfaces divided by the total inland surface per cell (NaturalEarthData.com, 1.24‐km resolution). One linear model was used per response variable (CTI, climatic debt, and CTI_SD_ trends). Fixed effects were the proportion of PA surface per cell and the temperature spatial shift, plus their interaction. To investigate the relationship between coverage of the studied PAs and their location in the western Palearctic, we also assessed with a linear model whether proportion of PA surface increased with latitude, longitude, and their interaction. Spatiotemporal changes in temperature, CTI, and climatic debt were expressed in units of kilometers per year and in degrees Celsius per year for the CTI_SD_. Each cell included both protected and unprotected sites and at least 15 sites (although the mean was substantially larger: 175 sites) to avoid cells with a small number of sites at the edge of the study area.

All statistical analyses were performed with R 3.4.3 (R Core Team 2017) with the glmmTMB package for the GLMMs and linear models (Magnusson et al. 2017).

## Results

### Protected Areas, CTI, CTI_SD_, and Climatic Debt

**Figure 2:**
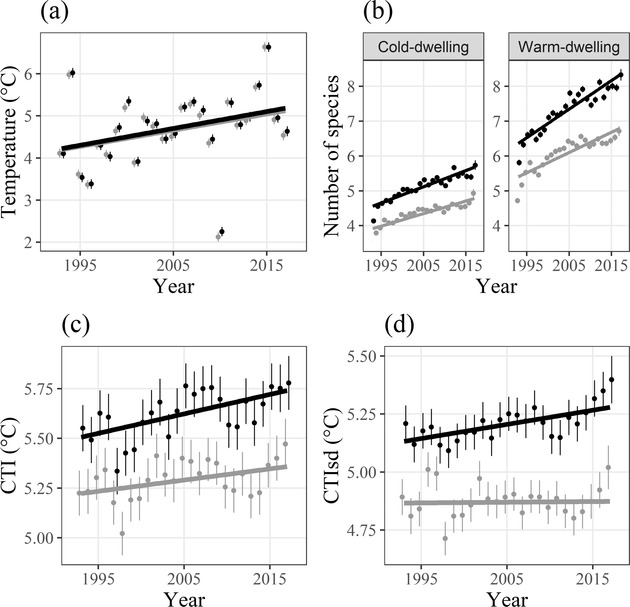
Temporal trends inside (black) and outside protected areas (gray) of (a) temperature, (b) number of cold‐ and warm‐dwelling species, (c) community temperature index (CTI), and (d) standard deviation of the CTI (CTI_SD_) (points, mean values; whiskers, 95% CI).

The temperature increased by 0.04° C/year (*p* < 0.001) without a significant difference between inside and outside PA (*p* = 0.2) (Table 1 & Fig. 2a). The CTI increased nearly twice as fast inside PAs than outside (approximately 0.010–0.006° C/year, respectively) (Table 1 & Fig. 2c). The CTI_SD_ increase was significant inside PAs, but not significant outside PAs (Table 1 & Fig. 2d). Therefore, inside PAs, the results matched scenario 3 (Fig. 1, colonization only), whereas outside PAs, the results matched scenario 4 (Fig. 1, colonization and extirpation). Complementary analyses based on a species resampling approach, on both species occurrence and abundance, confirmed the robustness of these results (Supporting Information).

**Table 1:** Parameter estimates of the temporal trends and main effects on temperature, community temperature index (CTI), standard deviation of the CTI (CTI_SD_), and number of cold‐ and warm‐dwelling species of site‐protection status (inside or outside protecteds area [PA]).*

Variable	Level	Main effects		Temporal trends (100 years)	
		estimate (SE)	post hoc t, p	estimate (SE)	post hoc t, p
Temperature	inside PA	4.69 (0.06)	–0.55, 0.58	3.99 (0.07)	–1.40, 0.16
	outside PA	4.65 (0.05)		3.86 (0.07)	
CTI	inside PA	5.62 (0.05)	–4.67, 0.001	0.98 (0.09)	–3.41, < 0.001
	outside PA	5.29 (0.05)		0.57 (0.08)	
CTI_SD_	inside PA	5.20 (0.03)	–8.58, < 0.001	0.61 (0.08)	–5.07, < 0.001
	outside PA	4.87 (0.03)		0.03 (0.08)	
Warm dwelling	inside PA	2.00 (0.01)	–11.16, 0.001	1.17 (0.02)	–5.98, < 0.001
	outside PA	1.81 (0.01)		0.97 (0.02)	
Cold dwelling	inside PA	1.64 (0.01)	–9.72, < 0.001	0.87 (0.03)	–3.06, 0.01
	outside PA	1.47 (0.01)		0.75 (0.03)	

* Temporal trends multiplied by 100 (i.e., trends for 100 years) to avoid decimals. For temperature, CTI, and CTI_SD_, df = 117, 319, and for cold and warm‐dwelling species, df = 203, 265.

Temporal changes in CTI lagged behind changes in temperature. The temperature latitudinal gradient was about −0.38° C/decimal degree (SE 0.005, z = −78.75, *p* < 0.001) and −0.31° C for the CTI (SE 0.004, z = −69.56, *p* < 0.001). The temperature increase was equivalent to a latitudinal shift of 11.4 km/year (285 km in 25 years). The temporal CTI trend was equivalent to a shift 43% larger inside PAs than outside (about 3.5 km/year inside PAs [87 km over 25 years] and 2.0 km/year outside [50 km over 25 years]). Consequently, the climatic debt was about 7.9 km/year inside PAs and 9.4 km/year outside (198 and 235 km over 25 years, respectively).

The number of species in the simplified cold‐dwelling and warm‐dwelling categories both increased significantly over the study period, but the trends and average numbers of species were significantly greater inside PAs (Table 1 & Fig. 2b). The number of warm‐dwelling species was higher (*β* = 0.346, *p* < 0.001) and increased faster than that of cold‐dwelling species (*β* = 0.003, *p* < 0.001) (Supporting Information). Inside PAs, the number of warm‐dwelling species also increased faster than that of cold‐dwelling species (Table 1). Our results suggest that based on 2 simplified thermal‐dwelling categories, the dynamic processes both inside and outside PAs were intermediate between scenarios 3 and 4 (i.e., more colonization than extirpation).

### Community Changes and Proportion of PA Surface

**Figure 3:**
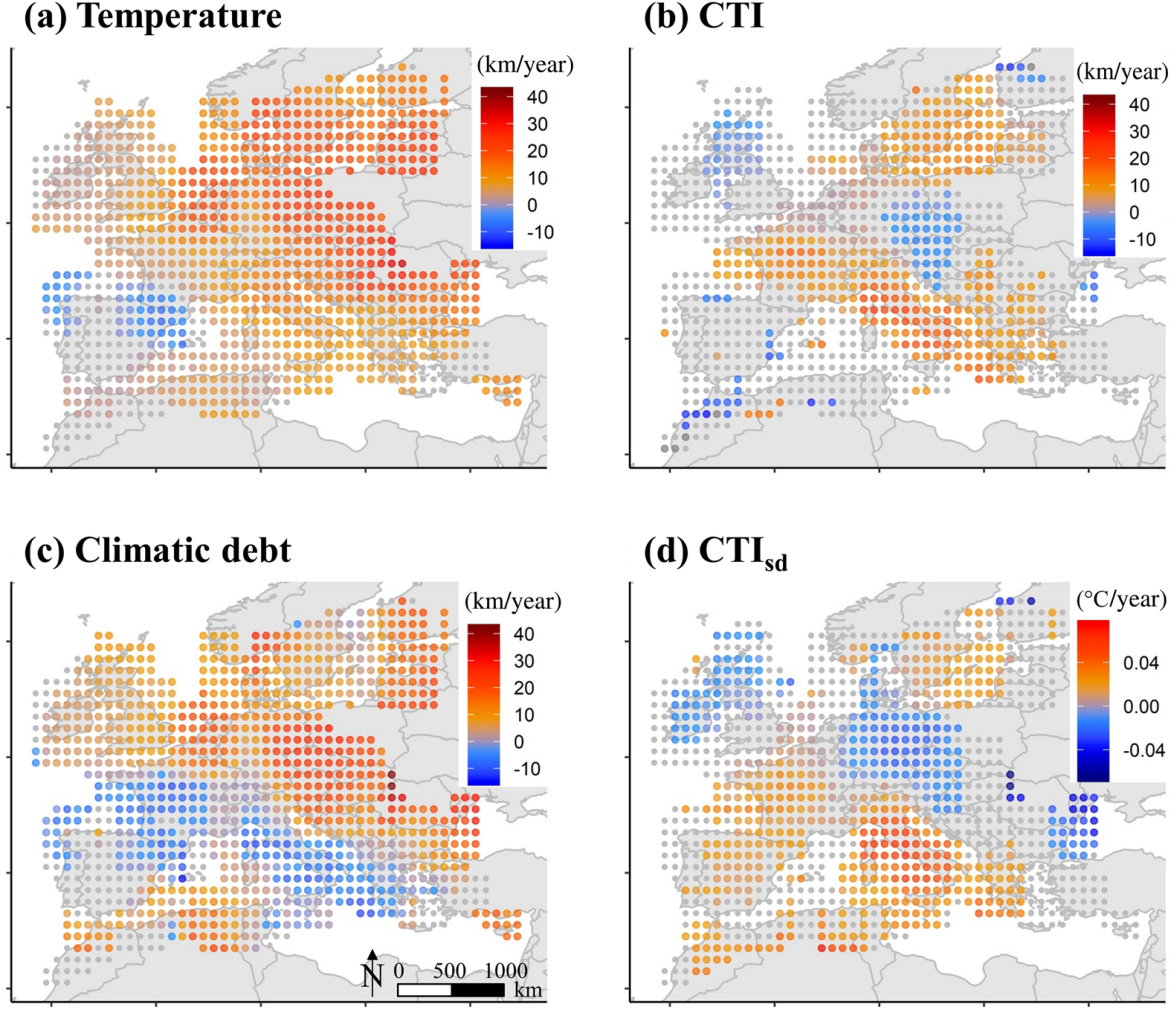
Spatiotemporal trends from 1993 to 2017 of (a) temperature, (b) community temperature index (CTI), (c) climatic debt, and (d) thermal heterogeneity (CTI_SD_) (points at centers of cells, trends [5° × 5° resolution]; red, positive trend, p < 0.05; blue, negative trend, p < 0.05; gray, not significant; color gradient, the darker the color, the greater the intensity).

The temperature increased significantly in 80% of the study area, with the exception of the northern half of the Iberian Peninsula (Fig. 3a). The CTI significantly increased in 37% of the cells (384/1032), mostly from the southern Balkans to western France and around the Baltic Sea (Fig. 3b). Consequently, there was climatic debt in 66% of the area, mostly in the northern half of Europe (Fig. 3c). Last, the CTI_SD_ trend was significantly positive in 39% of the cells, mainly in the east and the south, but also around the Baltic Sea (Fig. 3d).

The CTI spatial shift increased as inland proportion of PA surface increased per cell and temperature spatial shift increased (*p* ≤ 0.001); but the interaction of proportion of PA surface and temperature spatial shift was not significant (Table 2). Consequently, the climatic debts accumulated were smaller where there was a greater proportion of PA surface and greater where the temperature spatial shift was faster (*p* ≤ 0.001) (Table 2). The temporal trend of an increase in CTI_SD_ was smaller where the temperature spatial shift was faster (*p* < 0.001), but it was not significantly affected by the proportion of PA surface (Table 2). The proportion of PA surface was greater in northwest because the proportion of PA surface decreased with the longitude (*β* = −0.031, *p* < 0.001) and increased with the latitude (*β* = 0.011, *p* < 0.001). The interaction was negative and significant (*β* = −0.012, *p* < 0.001, Supporting Information).

**Table 2:** Parameter estimates of the spatial effect of proportion of inland protected area (PA) surface (log[e] transformed) and its interaction with the temperature spatial shift on the community temperature index (CTI) spatial shift, climatic debt, and CTI_SD_ per cell (5° × 5°)

Variable	Parameter	Coefficient	SE	z	p
CTI spatial shift	Intercept	3.092	0.203	15.259	< 0.001
	PA	0.505	0.208	2.430	0.015
	temperature spatial shift	1.131	0.203	5.570	< 0.001
	PA × temperature spatial shift	–0.160	0.216	–0.741	0.458
Climatic debt	Intercept	168.559	5.065	33.280	< 0.001
	PA	–12.620	5.193	–2.430	0.0151
	temperature spatial shift	165.667	5.072	32.640	< 0.001
	PA × temperature spatial Shift	4.007	5.404	0.740	0.458
CTI_SD_	Intercept	0.007	0.001	13.009	< 0.001
	PA	–0.001	0.001	–0.869	0.385
	temperature spatial shift	–0.005	0.001	–10.098	< 0.001
	PA × temperature spatial Shift	0.001	0.001	1.262	0.207

## Discussion

### Waterbird Community Adjustments to Climate Warming Inside PAs

Our study represents one of the first empirical and international assessments addressing differences in community changes in response to climate warming in PAs on a continental scale. We found that a fast increase in CTI inside PAs compared with outside PAs was driven mainly by colonization from warm‐dwelling species, which is consistent with other studies on birds and other taxonomic groups (Thomas et al. 2012; Gillingham et al. 2015). Indeed, when looking at finer spatial scale, the increase of CTI was more positive where the proportion of PA surface was larger, suggesting a positive relationship between PA coverage and community thermal changes (Gaüzère et al. 2016).

Overall, we found that the distribution changes of nonbreeding waterbirds in the western Palearctic showed a climatic debt, but that the debt was 16% lower inside PAs. Protected areas supported higher waterbird species richness than unprotected areas, which is consistent with the PA designation on wetlands of high biological importance under the international Ramsar Convention and the European Union's Nature Directives. Moreover, waterbird communities inside PAs had higher colonization, lower extirpation, and lower climatic debt than those outside PAs. These positive effects likely varied between PAs depending on how they are managed (Lawson et al. 2014). However, such conservation benefit is expected due to international conservation policies, which use PAs and species protection status as the main conservation measures to buffer the negative impacts of climate change (Trouwborst 2009). The western Palearctic falls under several of these international conventions, such as the Ramsar, Bern, and Bonn Conventions, and the benefits provided by habitat and species protection (Pavón‐Jordán et al. 2020) seem to effectively facilitate species’ adjustment to climate warming (Gaget et al. 2018).

Species richness of nonbreeding waterbirds increased over the study area, particularly inside PAs, in line with recent general positive trends of western Palearctic waterbird populations (Amano et al. 2018). Furthermore, inside––but not outside––PAs, the variation in CTI (i.e., CTI_SD_) increased over time, and we found a general increase in CTI of both cold‐ and warm‐dwelling species over time. Our findings suggest that inside PAs, species with high thermal affinity colonized the community, but at the same time, species with low thermal affinity were less likely to be extirpated, which likely increased their extinction debt. The PAs can act as refuges by improving species resilience against climate warming (Santangeli & Lehikoinen 2017), likely by ensuring ecological requirements needed for species persistence despite the proximity to their thermal niche edge. Consequently, despite smaller climatic debts inside PAs because of the large colonization by warm‐dwelling species, both persistence and colonization by cold‐dwelling species likely increased the climatic debt estimated for the whole community.

### Heterogeneity of Temperature and Community Changes

The intensity of the winter temperature warming increased along a southwest‐northeast gradient, driving the community adjustment through a similar gradient of intensity, although not perfectly (Fig. 3). The thermal isocline shift toward the northeast is related to the continental shape and the oceanic influence of the Gulf Stream (IPCC ). Interestingly, the nonsignificant temperature and CTI trends in the southwest western Palearctic resulted in negligible climatic debts. Conversely, the climatic debt increased in the northeastern countries, where strong temperature warming occurred (Fig. 3), which nonbreeding waterbirds were not be able to fully track.

Temperature was likely not the only aspect of the abiotic environment that influenced changes in species’ distribution. The local pattern of CTI changes contrasted with the expected relative increase of warm‐dwelling species. Although several other factors are likely to have affected species’ distribution changes, the CTI focuses on species assemblage changes in response to temperature changes, but its trend can also be affected by other drivers of population change (Bowler & Böhning‐Gaese 2017). For example, in the Untied Kingdom, despite a species‐specific west‐east waterbird redistribution (Austin & Rehfisch 2005), the CTI changes were likely altered by the recent increase of geese and the decrease of waders (Frost et al. 2019), which have low and high STIs, respectively (Supporting Information). Consequently, the subsequent community reshuffling may jeopardize the detection of a community thermal adjustment, if it exists (Bowler & Böhning‐Gaese 2017). Similarly, the absence of CTI increase in Central Europe and the Netherlands despite the temperature increase should encourage species‐specific investigations (Pavón‐Jordán et al. 2015). Such population changes, unexpected based on adjustment to climatic warming, may increase the mismatch between community and temperature changes (Galewski & Devictor 2016).

Although milder climate conditions reduce ice and snow in the northern and eastern regions and enhance northward range expansion (Brommer 2008; Schummer et al. 2010; Pavón‐Jordán et al. 2019), community adjustment to climate warming was not particularly strong in northern Europe (Fig. 3). This may be the result of average temperatures not accurately reflecting the thermal conditions that affect changes in species’ distribution. For example, in the northern regions, severe cold spells may potentially cause high mortality events, thus limiting species distribution changes (Pavón‐Jordán et al. 2019).

Considering the strong waterbird distribution change in northern Europe (Brommer 2008; Lehikoinen et al. 2013), the lack of CTI increase also suggests some limits of the CTI framework. The CTI measures changes in species assemblages (Devictor et al. 2008) and could be sensitive to the number of species already present in the community. Indeed, when there are few species at the beginning of the monitoring, because of ice cover for example, the CTI trend should be more sensitive to the appearance of new species. We did not take this potential uncertainty into account. Consequently, our ability to measure species distribution change was challenged in these ice‐dominated regions, where the community adjustment to climate warming is likely underestimated (Fox et al. 2019).

### Perspectives for Research and Conservation

Indicators are essential tools to synthesize population dynamics and inform public policies (Tittensor et al. 2014). The CTI is an intuitive indicator with which to measure and communicate the impact of climate warming on communities (Devictor et al. 2012; Gaüzère et al. 2019). Here, we went one step farther and used the CTI_SD_ to identify the colonization‐extirpation patterns in response to climate warming (Supporting Information). With these simple indicators, we identified that the community adjustment to temperature was mainly due to high colonization by the warm‐dwelling and reduced extirpation of cold‐dwelling species inside PAs, whereas outside PAs, the extirpation of the most cold‐dwelling species was nearly equivalent to the colonization by warm‐dwelling species (Fig. 2d).

We reliance on an internationally coordinated monitoring program, which allowed us to investigate whether community adjustment to climate warming was higher in PAs. The IWC is a monitoring scheme that aims to ensure waterbird counts (full checklists) in both protected and unprotected areas (Delany 2010). However, PAs were not randomly distributed (Supporting Information) and such nonrandomness could induce spatial aggregation between PA density and CTI changes. Nevertheless, when looking at the spatiotemporal changes (Fig. 3), spatial aggregation was moderate. Also, because the CTI is an index summarizing the community of species, it may be sensitive to false absences of species (i.e., species that were present but not detected). Occupancy models provide a framework for correcting for false absences, but the design of the IWC data‐collection protocol does not include the recording of information on the factors that could affect detection rates (e.g., observer, time and land cover) that is necessary for the modeling of variation in detection rates. Although our estimates of CTI scores contained errors, we do not believe these errors caused biases that affect our conclusions. Each species can be cold or warm dwelling relative to the other species in the context of different communities; thus, species‐specific differences in detectability should not affect the differences in CTI trends inside and outside PAs at the western Palearctic scale.

Nonbreeding waterbirds have high capacity to respond to climate warming with a distribution change (Maclean et al. 2008; Lehikoinen et al. 2013; Pavón‐Jordán et al. 2019), even more than other groups of birds (Brommer 2008). Our study reveals a relatively fast average distribution shift, 2.0–3.5 km/year, which is greater than rates reported for the European common breeding birds (2.1 km/year [Devictor et al. 2012]) and other taxa (1.8 km/year [Chen et al. 2011]). Indeed, because most of the western Palearctic waterbirds are migratory, overwintering at more northern latitudes could be advantageous for them because migration cost would be lower, which benefits their fitness (Reneerkens et al. 2019).

The rapid distributional changes that we found bring into question the future effectiveness of the PA networks because the locations of these sites potentially do not match the future distributions of waterbird species (Araújo et al. 2004). In the western Palearctic, even if the number of PAs increases in the north, the network still does not cover all the wetlands important for waterbird conservation (Pavón‐Jordán et al. 2015; Guillemain & Hearn 2017; 2020). More studies are needed to evaluate the current and future coherence and cohesiveness of the PA network, particularly for species of conservation concern.

## Supporting information

Additional information is available online in the Supporting Information section at the end of the online article. The authors are solely responsible for the content and functionality of these materials. Queries (other than absence of the material) should be directed to the corresponding author.
