# Supplementary material for: Benefits of protected areas for nonbreeding waterbirds adjusting their distributions under climate warming
Source: Conserv Biol. 2021 Jan 21;35(3):834–45. doi: 10.1111/cobi.13648 (PMC8247957; doi:10.1111/cobi.13648)

**Non-breeding waterbirds benefit from protected areas when adjusting their distribution to climate warming**

Appendix 1. Colonization and extinction patterns revealed by the CTI_sd_

*1. Simulations of the species extinction/colonization in response to temperature increase and subsequent changes of Community Temperature Index (CTI) and standard deviation (CTI_sd_) over time.*

Following the Figure 1, four scenarios were simulated (Rcode below). The scenarios were: (1) ’No colonization-No extinction’; (2) ‘Extinction only’; (3) ‘Colonization only’; (4) ‘Colonization-Extinction’.

For each of the four scenarios, we simulated an occurrence matrix for 100 species considered in three temperature dwelling classes over 25 years (from 1 to 25) and 100 sites. We attributed different Species Temperature Index (STI) values to the species from a random simulation of STI values based on a Gaussian distribution of mean 0 and SD 10. Twenty five species were considered as extreme cold-dwelling species with STI inferior to -5°C, 50 species were considered as slight cold- or warm-dwelling species with STI between -5°C and 5°C and 25 species were considered as extreme warm dwelling species with STI superior to 5°C. Species occurrence were simulated from a binomial distribution with different probabilities between the extreme cold-dwelling (p=0.25 or p=0.25-year/100, if extinction), slight cold- or warm-dwelling species (p=0.75) and extreme warm-dwelling species (p=0 or p=year/100, if colonization). From the 100 original species pool, 1 to 90 species were randomly removed in order to simulated different environmental filters. We computed the CTI and CTI_sd_ values per year per site (see Methods). We used generalized linear mixed effects models (GLMM, Gaussian error distribution) with the CTI or CTIsd as the response variable, the year as the explanatory term and the site in random effect. Finally, the estimate temporal slope and its p-value were collected. We simulated the four scenarios 100 times following this process (Rcode below).

Rcode used for the simulations:

library(glmmTMB);library(dplyr);library(effects);library(ggplot2);library(ggpubr)

CTIcalc<-function(x){sum(log(x+1)*STI/sum(log(x+1)))}

CTIsdcalc_occ<-function(x){ a<-STI*x;a[a==0]<-NA;sd(a,na.rm=T)}

M_CTItot=as.data.frame(matrix(nr=4,nc=2));M_CTIsd=as.data.frame(matrix(nr=4,nc=2))

M_Simul_CTItot=NULL;M_Simul_CTIsd=NULL

**# STI simulations**

for(s in 1:100){

STI<-as.data.frame(sort(rnorm(1000, mean = 0, sd = 10)))

STIwarm<-sort(sample(STI[STI>5], 25))

STImid<-sort(sample(STI[STI>(-5)&STI<5], 50))

STIcold<-sort(sample(STI[STI<(-5)], 25))

L_model_occ<-list()

for(model in 1:4){

warm=cold<-as.data.frame(matrix(nc=25,nr=25*100))

mid<-as.data.frame(matrix(nc=50,nr=25*100))

colnames(warm)<-rep(paste0("warm",1:25)); colnames(mid)<-rep(paste0("mid",1:50));colnames(cold)<-rep(paste0("cold",1:25))

mid$site<-warm$site<-cold$site<-sort(rep(paste0("site",1:100),25))

mid$year<-warm$year<-cold$year<-rep(1993:2017,100)

site<-unique(warm$site)

tempo_mid=tempo_warm=tempo_cold<-NULL

**#Cold-dwelling species occurrence**

tempo<-NULL

for(i in 1:100){

tempo<-subset(cold[,1:25],cold$site==site[i])

for(k in 1:25){

for(j in 1:25){

proba_cold<-c(0.25,(0.25-j/100),0.25,(0.25-j/100))

proba<-proba_cold[model]

proba[proba<0]<-0

tempo[j,k]<-rbinom(1,1,prob=proba)

} }

tempo_cold<-rbind(tempo_cold,tempo) }

**#Slight cold- and warm-dwelling species occurrence**

tempo<-NULL

for(i in 1:100){

tempo<-subset(mid[,1:50],mid$site==site[i])

for(k in 1:50){

for(j in 1:25){

tempo[j,k]<-rbinom(1,1,prob=0.75)

} } tempo_mid<-rbind(tempo_mid,tempo) }

**#Warm-dwelling species occurrence**

tempo<-NULL

for(i in 1:100){

tempo<-subset(warm[,1:25],warm$site==site[i])

for(k in 1:25){

for(j in 1:25){

proba_warm<-c(0,0,(j/100),(j/100))

proba<-proba_warm[model]

proba[proba<0]<-0

tempo[j,k]<-rbinom(1,1,prob=proba)

} }

tempo_warm<-rbind(tempo_warm,tempo) }

**#Random species exclusion**

warm[,1:25]<-tempo_warm;tab_cti1<-as.data.frame(cbind(tempo_cold,tempo_mid,warm))

tab_cti=tempo=NULL

for(i in 1:100){

tempo<-subset(tab_cti1[,1:100],tab_cti1$site==site[i])

tempo[,c(sample(1:100,sample(1:90,1),replace=F))]<-0

tab_cti<-rbind(tab_cti,tempo) }

**#Model**

tab_cti$site<-tab_cti1$site;tab_cti$year<-tab_cti1$year

STI<-c(STIcold,STImid,STIwarm)

tab_cti$CTItot<-apply(tab_cti[,1:100],1,CTIcalc); tab_cti$CTIsd<-apply(tab_cti[,1:100],1,CTIsdcalc_occ)

L_model_occ[[model]]<-tab_cti

CTI_year<-glmmTMB(CTItot~year+(1|site), family=gaussian(link = "identity"),data=L_model_occ[[model]])

CTI_year_sd<-glmmTMB(CTIsd~year+(1|site), family=gaussian(link = "identity"),data=L_model_occ[[model]])

M_CTItot[model,1:2]<-summary(CTI_year)$coefficients$cond[c(2,8)]

M_CTIsd[model,1:2]<-summary(CTI_year_sd)$coefficients$cond[c(2,8)]}

M_Simul_CTItot<-rbind(M_Simul_CTItot,M_CTItot)

M_Simul_CTIsd<-rbind(M_Simul_CTIsd,M_CTIsd) }

M_Simul_CTI<-as.data.frame(cbind(M_Simul_CTItot,M_Simul_CTIsd))


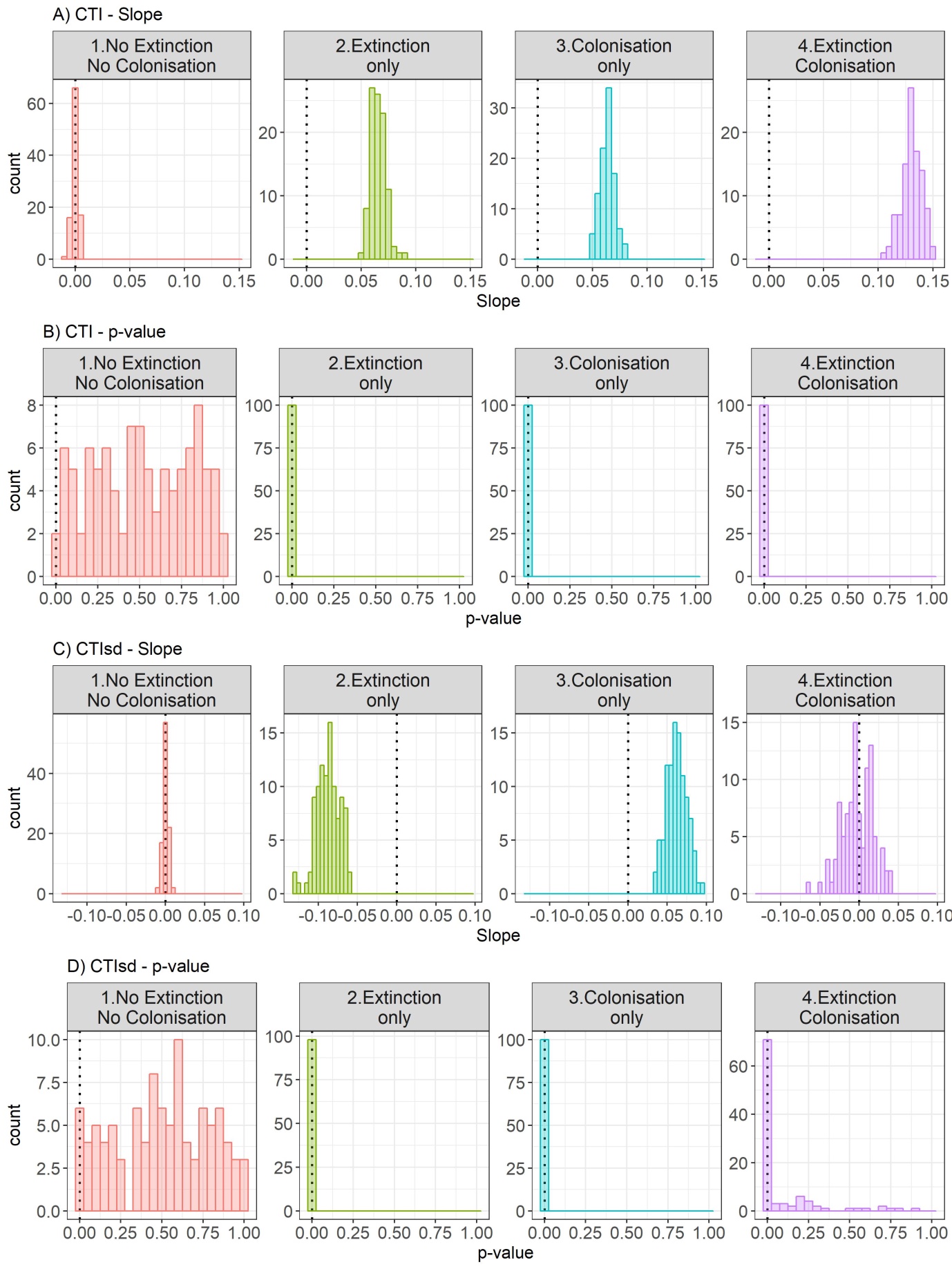


Figure S1a: Histograms of the model outputs per scenario. A) CTI estimated slope, B) p-value corresponding to the CTI slope, C) CTIsd estimated slope, D p-value corresponding to the CTIsd slope. The scenarios of community changes in response to temperature increase were: (1) ’No colonization-No extinction’; (2) ‘Extinction only’; (3) ‘Colonization only’; (4) ‘Colonization-Extinction’.

2. *Empirical observation of waterbird species extinction/colonization in response to temperature increase and subsequent changes of Community Temperature Index average (CTI) and standard deviation (CTI_sd_) over time.*

We highlighted the ability of the CTI_sd_ to be an indicator of colonization and extinction processes in response to climate warming. Indeed, community changes in response to temperature increase should result in four scenarios: (1) ’No colonization-No extinction’ causes no CTI and CTI_sd_ changes; (2) ‘Extinction only’ causes CTI increase and CTI_sd_ decrease by the loss of cold-dwelling species; (3) ‘Colonization only’ causes CTI and CTI_sd_ increase by the gain of warm-dwelling species; (4) ‘Colonization-Extinction’ causes CTI increase by the species thermal turn-over, but no CTI_sd_ directional change (Fig. 1). We classified the count events in the four scenarios of colonization and/or extinction events, following what happening between the monitoring year and the next one (e.g., if between the counts *i* and *i+1* only one species colonized the site, the count *i* correspond to the scenario (3) ‘Colonization only’). For each count event, we measure the change of CTI_sd_ from a monitoring year and the next one (i.e., ΔCTI_sd_), which is supposed to be superior, inferior or equal to zero depending of the four colonization/extinction scenarios in response to temperature increase. We used a GLMM per scenarios (Gaussian error distribution) to investigate if the ΔCTI_sd_ values correspond to the expected patterns following the four scenarios. Site was added in random factors.

Conformely to the expectation under a community adjustment to climate warming, the ΔCTI_sd_ values was null in case of no extinction and no colonization (β = 0.000, P = 1), significantly negative in case of extinction only (β = -0.582, P < 0.001), significantly positives in case of colonization only (β = 0.590, P < 0.001), and not significantly different from zero in case of extinction and colonization (β = -0.005, P = 0.5) (Fig. S1b).

*
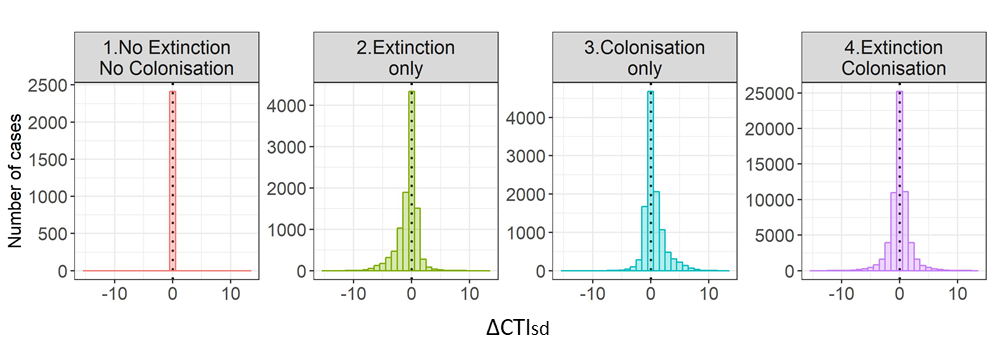
*

Figure S1b: Histograms of the ΔCTI_sd_ values over the four scenarios of community changes in response to climate warming: (1) ’No colonization-No extinction’ causes no CTI_sd_ changes, (2) ‘Extinction only’ causes CTI_sd_ decrease by the loss of cold-dwelling species, (3) ‘Colonization only’ causes CTI_sd_ increase by the gain of warm-dwelling species, (4) ‘Colonization-Extinction’ causes no CTI_sd_ directional change (Fig. 1). The dotted line is positioned on the zero to signify the absence of CTI_sd_ change.

Appendix 2. Map of the study area and details of the monitoring per country.


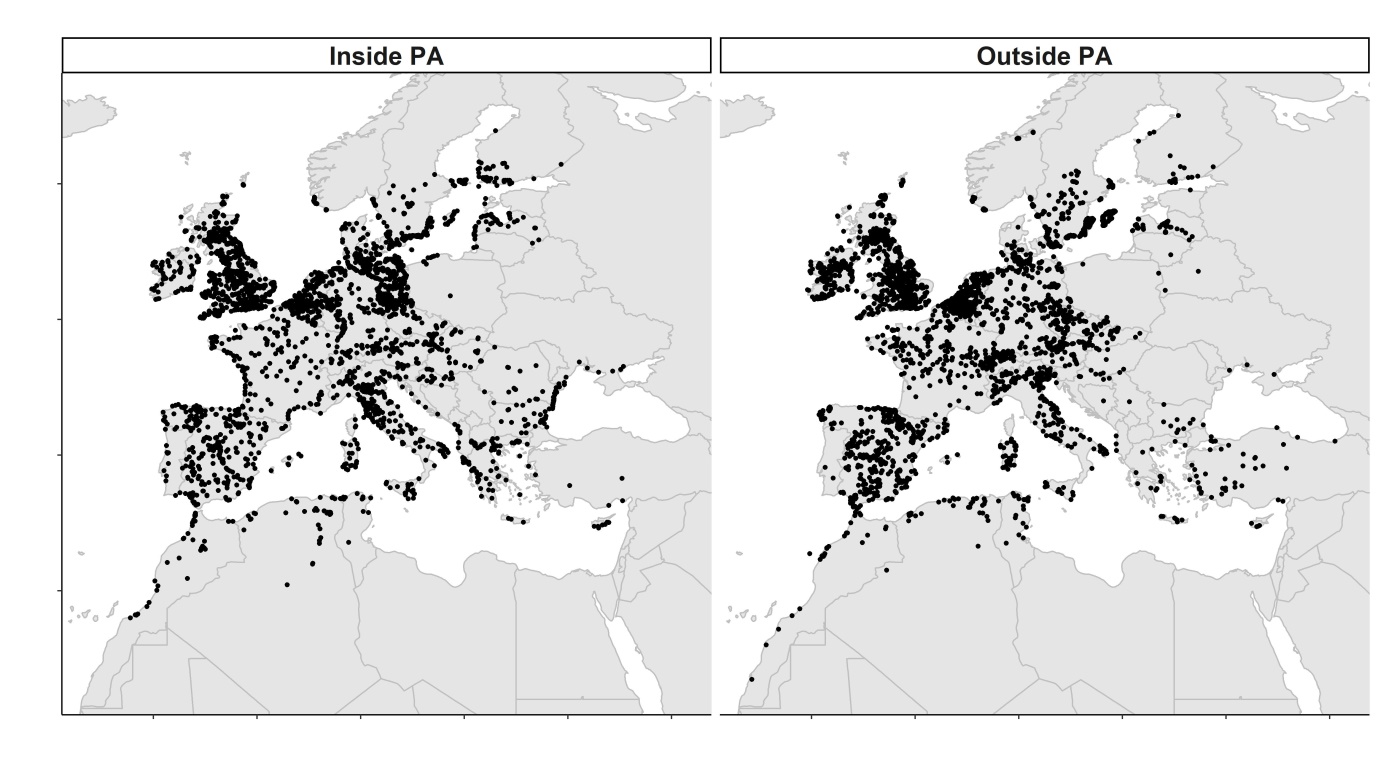


Figure S2: Map of the study area including 7,071 monitoring sites inside a protected area (PA, n = 3,374) and outside (n = 3,697), in 39 Western-Palearctic countries. The sites inside and outside PAs had a similar number of counts (in average [SD] 16.8±5.7 and 16.4±5.7, respectively) and a similar spatial distribution (in average [SD] Lat. 49.8±6.2, Lon. 7.0±9.1 and Lat. 50.3±6.1, Lon. 5.2±9.0, respectively.

Table S2: Details of the monitoring per country including per country, the number of monitored sites, the average number of surveys, the average period covered by the survey (in year), the number of sites included in a protected area, the total waterbird abundance and the change of monitoring change (see Appendix 4).

| Country | Number of sites | Average number of surveys | Average covered period | Number of sites included in a protected area | Total waterbird abundance (million) | Monitoring changes |
| --- | --- | --- | --- | --- | --- | --- |
| Albania | 13 | 14.6 | 22.8 | 10 | 2.67 | No |
| Algeria | 75 | 15.7 | 22.0 | 42 | 4.08 | No |
| Austria | 174 | 20.4 | 22.8 | 68 | 2.76 | No |
| Belarus | 4 | 8.3 | 20.5 | 1 | 0.10 | No |
| Belgium:Flanders | 472 | 19.8 | 22.6 | 234 | 5.83 | Yes |
| Belgium:Wallonia | 180 | 13.0 | 15.9 | 48 | 0.71 | No |
| Bosnia & Herzegovina | 1 | 11.0 | 16.0 | 0 | 0.02 | No |
| Bulgaria | 46 | 18.7 | 22.2 | 36 | 6.90 | No |
| Croatia | 32 | 11.5 | 21.5 | 26 | 1.50 | No |
| Cyprus | 12 | 15.8 | 22.4 | 8 | 0.27 | No |
| Czechia | 215 | 15.7 | 22.3 | 60 | 2.38 | No |
| Denmark | 48 | 22.1 | 23.5 | 35 | 6.21 | Yes |
| Estonia | 4 | 19.0 | 19.5 | 3 | 0.06 | No |
| Finland | 172 | 15.9 | 21.5 | 101 | 0.89 | No |
| France | 291 | 22.3 | 23.3 | 137 | 52.60 | No |
| Germany | 1057 | 15.6 | 19.1 | 749 | 35.02 | No |
| Greece | 92 | 13.0 | 19.2 | 72 | 8.26 | No |
| Hungary | 25 | 17.1 | 20.9 | 19 | 2.91 | No |
| Ireland | 255 | 14.4 | 19.3 | 61 | 6.02 | Yes |
| Italy | 458 | 17.9 | 20.7 | 241 | 32.84 | No |
| Latvia | 144 | 13.5 | 22.1 | 71 | 0.82 | No |
| Lithuania | 5 | 13.6 | 22.2 | 4 | 0.78 | No |
| Montenegro | 1 | 22.0 | 24.0 | 1 | 2.90 | No |
| Morocco | 65 | 11.9 | 21.6 | 36 | 6.66 | No |
| Netherlands | 173 | 23.8 | 22.9 | 59 | 98.62 | No |
| North Macedonia | 2 | 13.5 | 20.0 | 2 | 0.55 | No |
| Norway | 57 | 16.6 | 23.5 | 28 | 0.36 | No |
| Poland | 12 | 10.5 | 21.5 | 10 | 0.22 | No |
| Portugal | 13 | 17.3 | 22.5 | 11 | 2.66 | No |
| Romania | 34 | 11.1 | 17.5 | 34 | 1.75 | Yes |
| Serbia | 2 | 5.5 | 20.5 | 0 | 0.07 | No |
| Slovakia | 80 | 10.8 | 14.5 | 39 | 0.82 | No |
| Slovenia | 2 | 16.5 | 20.5 | 2 | 0.04 | No |
| Spain | 780 | 13.5 | 19.2 | 351 | 23.63 | No |
| Sweden | 705 | 17.6 | 22.7 | 243 | 8.64 | Yes |
| Switzerland | 100 | 22.6 | 23.2 | 24 | 13.40 | No |
| Tunisia | 31 | 10.9 | 22.9 | 11 | 3.27 | No |
| Turkey | 45 | 7.8 | 19.1 | 5 | 8.65 | No |
| Ukraine | 11 | 9.7 | 17.7 | 8 | 1.70 | No |
| United Kingdom | 1615 | 18.0 | 21.1 | 660 | 50.07 | Yes |

Appendix 3. Additional species information.

The winter STI is the long-term average January temperature (WorldClim database, 1950-2000, http://worldclim.org/) experimented by the species across its non-breeding (overwintering) distribution (extracted from www.birdlife.org 2015) only inside the African-Eurasian region defined by the African-Eurasian Migratory Waterbird Agreement (AEWA, http://www.unep-aewa.org). We removed the distribution of the sub-species resident in sub-Saharan Africa to avoid an overestimation of the thermal affinity tolerated by the studied populations (Involved species: *Ardea alba, Ardea cinerea, Botaurus stellaris, Gallinula chloropus, Phalacrocorax carbo, Podiceps cristatus, Podiceps nigricollis, Porphyrio porphyrio* and *Tachybaptus ruficollis*). Species considered as vagrant when their overwintering distribution was not included in the AEWA area and in the Western-Palearctic with a minimum threshold of 500 individuals over the 25 years.

Table S3: List of the species with their species temperature index (STI) and the number of sites occupied at least once.

| Scientific name | STI | Number of sites occupied |
| --- | --- | --- |
| *Actitis hypoleucos* | 23.16 | 1274 |
| *Anas acuta* | 16.90 | 2696 |
| *Spatula clypeata* | 14.91 | 4929 |
| *Anas crecca* | 12.72 | 6854 |
| *Mareca penelope* | 16.53 | 1642 |
| *Anas platyrhynchos* | -0.02 | 3770 |
| *Mareca strepera* | 11.73 | 734 |
| *Anser albifrons* | 2.53 | 93 |
| *Anser anser* | 4.47 | 1229 |
| *Anser brachyrhynchus* | 2.02 | 2246 |
| *Anser erythropus* | 2.43 | 6239 |
| *Anser fabalis & serrirostris* | -2.51 | 586 |
| *Ardea alba* | 5.41 | 4994 |
| *Ardea cinerea* | 4.32 | 5303 |
| *Arenaria interpres* | 17.86 | 1696 |
| *Aythya ferina* | 11.34 | 744 |
| *Aythya fuligula* | 10.45 | 857 |
| *Aythya marila* | 0.43 | 577 |
| *Aythya nyroca* | 9.77 | 1444 |
| *Botaurus stellaris* | 17.73 | 182 |
| *Branta bernicla* | 2.86 | 853 |
| *Branta leucopsis* | 1.69 | 4057 |
| *Branta ruficollis* | 1.10 | 539 |
| *Bubulcus ibis* | 22.96 | 1297 |
| *Bucephala clangula* | -1.11 | 420 |
| *Calidris alba* | 18.86 | 254 |
| *Calidris alpina* | 11.83 | 467 |
| *Calidris canutus* | 19.08 | 550 |
| *Calidris maritima* | -2.48 | 498 |
| *Calidris minuta* | 22.77 | 697 |
| *Charadrius alexandrinus* | 19.20 | 630 |
| *Charadrius hiaticula* | 22.11 | 1103 |
| *Ciconia ciconia* | 22.95 | 707 |
| *Clangula hyemalis* | -2.50 | 2258 |
| *Cygnus columbianus* | 2.44 | 5300 |
| *Cygnus cygnus* | -1.49 | 1796 |
| *Cygnus olor* | 1.27 | 5837 |
| *Egretta garzetta* | 21.26 | 65 |
| *Fulica atra* | 5.86 | 2653 |
| *Fulica cristata* | 22.59 | 4535 |
| *Gallinula chloropus* | 5.61 | 862 |
| *Gallinago gallinago* | 18.58 | 944 |
| *Gavia arctica* | 0.76 | 581 |
| *Gavia stellata* | 3.05 | 949 |
| *Grus grus* | 14.72 | 379 |
| *Haematopus ostralegus* | 15.34 | 549 |
| *Himantopus himantopus* | 22.13 | 4140 |
| *Hydrocoloeus minutus* | 3.70 | 172 |
| *Larus argentatus* | 4.58 | 3413 |
| *Ichthyaetus audouinii* | 11.45 | 2046 |
| *Larus canus* | 1.62 | 207 |
| *Larus fuscus* | 18.58 | 1707 |
| *Chroicocephalus genei* | 11.90 | 607 |
| *Larus marinus* | -2.49 | 4695 |
| *Ichthyaetus melanocephalus* | 9.31 | 478 |
| *Chroicocephalus ridibundus* | 6.58 | 596 |
| *Limosa lapponica* | 19.23 | 4323 |
| *Limosa limosa* | 21.01 | 3760 |
| *Marmaronetta angustirostris* | 5.95 | 87 |
| *Melanitta fusca* | 0.67 | 904 |
| *Melanitta nigra* | 2.28 | 838 |
| *Mergellus albellus* | -1.58 | 2396 |
| *Mergus merganser* | -0.40 | 3945 |
| *Mergus serrator* | -1.08 | 1911 |
| *Microcarbo pygmeus* | 2.74 | 286 |
| *Netta rufina* | 5.35 | 1208 |
| *Numenius arquata* | 18.89 | 1738 |
| *Numenius phaeopus* | 22.06 | 228 |
| *Nycticorax nycticorax* | 23.47 | 218 |
| *Oxyura leucocephala* | 1.27 | 168 |
| *Pelecanus crispus* | 8.37 | 116 |
| *Pelecanus onocrotalus* | 22.01 | 71 |
| *Phalacrocorax aristotelis* | 2.30 | 499 |
| *Phalacrocorax carbo* | 3.70 | 6160 |
| *Philomachus pugnax* | 22.96 | 465 |
| *Phoenicopterus roseus* | 20.58 | 440 |
| *Platalea leucorodia* | 17.95 | 192 |
| *Plegadis falcinellus* | 23.14 | 1269 |
| *Pluvialis apricaria* | 5.36 | 692 |
| *Pluvialis squatarola* | 18.84 | 742 |
| *Podiceps auritus* | 3.39 | 4742 |
| *Podiceps cristatus* | 3.25 | 798 |
| *Podiceps grisegena* | 2.27 | 1256 |
| *Podiceps nigricollis* | 6.63 | 253 |
| *Porphyrio porphyrio* | 8.20 | 1810 |
| *Rallus aquaticus* | 4.52 | 563 |
| *Recurvirostra avosetta* | 22.04 | 1094 |
| *Somateria mollissima* | -7.53 | 3114 |
| *Tachybaptus ruficollis* | 3.54 | 4917 |
| *Tadorna ferruginea* | 8.88 | 500 |
| *Tadorna tadorna* | 4.30 | 2444 |
| *Sterna sandvicensis* | 12.38 | 422 |
| *Tringa erythropus* | 21.03 | 473 |
| *Tringa nebularia* | 23.16 | 748 |
| *Tringa ochropus* | 21.10 | 1475 |
| *Tringa totanus* | 15.48 | 1412 |
| *Vanellus vanellus* | 4.52 | 2933 |

Appendix 4: Additional monitoring information and CTI correction.

The International Waterbird Census (IWC) started for some species in the 1960s, but had comprehensive species coverage by the end of the 1980s. To be cautious, we started the study period in 1993. However, in some countries gulls and shags were not included directly in the IWC. The full waterbird species census was performed later in Romania (1999), Belgium (Flandre, 2000), Denmark (2001), United Kingdom (2002), Ireland (2002) and Sweden (still not full). As a change in species monitored can artificially affect the community changes, we took these dates into account in the analyses.

The community temperature index (CTI) was corrected to account for the monitoring changes in countries where the full waterbird species census started after the beginning of the study period (countries listed above). In these countries, the CTI values before the year(s) of monitoring change were centred per site (not reduced) and added to the average site CTI value of the years after the monitoring change. Hence, the addition of new species after the monitoring change doesn’t strongly affect the CTI values (Appendix 4, Table S1). Note that under the hypothesis of a CTI increase over years, the CTI correction leads to an overestimation of the site CTI average before the monitoring change. Regarding the CTI_sd_ no adaptation was done.

Table S4: Summary of the variance minimum (Min.) median, mean and maximum (Max.) between the original CTI computed without and with correction.

| Variable | Min. | Median | Mean | Max. |
| --- | --- | --- | --- | --- |
| CTI uncorrected | -5.014 | 5.392 | 5.514 | 22.544 |
| CTI corrected | -5.014 | 5.382 | 5.516 | 22.544 |

We performed models with the full dataset and the data subset to control the potential differences. We used the same model framework as in the Methods section to evaluate the change of CTI, CTI_sd_, number of cold-dwelling species and number of warm-dwelling species. As a result, the models outputs were fairly similar between the two dataset, at the exception that warm-dwelling species did not significantly increased more than cold-dwelling species inside PAs (Appendix 4, Table S2).

Table S2: Comparison of the models with the full dataset and the subset of data with the full species monitoring (1993-2017). Parameter estimates of the temporal trends of protected area (PA) effect on the community temperature index (CTI) and standard deviation of the CTI (CTI_sd_), number of cold- and warm-dwelling species. Base line is sites outside PA in all models and cold-dwelling species in the thermal-dwellers models. Interactions are notified by ‘:’ in all models and Years were standardized to zero mean (std.) in the thermal-dwellers models.

| Dataset | Variable | Parameter | Coefficient | SE | Z-value | P-value |
| --- | --- | --- | --- | --- | --- | --- |
| Full dataset | CTI | Intercept | -6.030 | 1.696 | -3.554 | < 0.001 |
|  |  | Year | 0.006 | 0.001 | 6.676 | < 0.001 |
|  |  | PA | 7.972 | 2.435 | 3.274 | < 0.001 |
|  |  | Year:PA | 0.004 | 0.001 | 3.412 | < 0.001 |
|  | CTI_sd_ | Intercept | 4.294 | 1.602 | 2.680 | 0.007 |
|  |  | Year | 0.000 | 0.001 | 0.360 | 0.719 |
|  |  | PA | 11.310 | 2.300 | 4.920 | < 0.001 |
|  |  | Year:PA | 0.006 | 0.001 | 5.066 | < 0.001 |
|  | Cold- and Warm-dwellers | Intercept | 1.466 | 0.012 | 125.070 | < 0.001 |
|  |  | Year(std.) | 0.057 | 0.002 | 33.560 | < 0.001 |
|  |  | PA | 0.163 | 0.017 | 9.600 | < 0.001 |
|  |  | Dweller | 0.338 | 0.002 | 154.600 | < 0.001 |
|  |  | Year(std.):PA | 0.005 | 0.002 | 2.360 | 0.018 |
|  |  | Year(std.):Dweller | 0.008 | 0.002 | 3.820 | < 0.001 |
|  |  | PA:Dwellers | 0.022 | 0.003 | 7.360 | < 0.001 |
|  |  | Year(std.):PA:Dweller | 0.006 | 0.003 | 1.960 | 0.050 |
| Subset with full species monitoring | CTI | Intercept | 0.098 | 2.241 | 0.044 | 0.965 |
|  |  | Year | 0.003 | 0.001 | 2.563 | 0.010 |
|  |  | PA | 15.302 | 3.123 | 4.900 | < 0.001 |
|  |  | Year:PA | 0.008 | 0.002 | 4.930 | < 0.001 |
|  | CTI_sd_ | Intercept | 2.396 | 2.051 | 1.168 | 0.243 |
|  |  | Year | 0.001 | 0.001 | 1.290 | 0.197 |
|  |  | PA | 8.743 | 2.858 | 3.059 | 0.002 |
|  |  | Year:PA | 0.004 | 0.001 | 3.153 | 0.002 |
|  | Cold- and Warm-dwellers | Intercept | 1.473 | 0.012 | 124.750 | < 0.001 |
|  |  | Year(std.) | 0.049 | 0.002 | 26.090 | < 0.001 |
|  |  | PA | 0.166 | 0.017 | 9.720 | < 0.001 |
|  |  | Dweller | 0.334 | 0.002 | 141.280 | < 0.001 |
|  |  | Year(std.):PA | 0.008 | 0.003 | 3.060 | 0.002 |
|  |  | Year(std.):Dweller | 0.014 | 0.002 | 6.020 | < 0.001 |
|  |  | PA:Dwellers | 0.024 | 0.003 | 7.460 | < 0.001 |
|  |  | Year(std.):PA:Dweller | 0.005 | 0.003 | 1.620 | 0.104 |

Appendix 5: CTI and CTI_sd_ trend robustness

We did complementary analyse to assess the robustness of the results to the species identity and abundance, using a re-sampling approach following Devictor et al. (2012) in which the CTI and CTI_sd_ trends were estimated after the random removal of 20% of the species (1000 iterations), using both occurrence and abundance.

We first computed CTI and CTI_sd_ on species occurrence (presence/absence) and abundance, removing randomly 20% of the species 1000 times to control whether the results rely on few influential species. The CTI(occ) based on occurrence is the average species temperature index (STI) of the species present in the community per count event. The CTI(ab) based on abundance corresponds to the average of the species STI weighed by the log(abundance+1) of the species (the log is used to buffer the influence of the highly abundant waterbird species as in Godet et al. (2011)). The CTI_sd_(occ) is the standard deviation of the species STI present in the community per count event, weighed by the log(abundance+1) for the CTI_sd_(ab).

We assessed the temporal trends and effects of site protection status (PA) on CTI(occ), CTI(ab), CTI_sd_(occ) and CTI_sd_(ab) using 1000 generalized linear mixed effects models (GLMM, Gaussian error distribution) per variable. The explanatory terms were the *year* (continuous variable from 1993-2017), the site *protection status* (Inside or Outside) and the interaction *year* × *protected status*. The *site identity* was added as a random effect and the spatial autocorrelation was taken into account by including the site geographical coordinates as an exponential spatial correlation structure in the model.

Overall, the distributions of the estimates assessed without 20% of the species were fairly normaly distributed and very rarely overlapping zero (Figure S3). In addition, the estimates based on all the data (blue solid lines) fall roughly in the middle of the histograms. This suggests that the results are consistent between occurrence and abundance (same patterns and order of magnitude) and not influenced by a few species only. Indeed, the trends inside PA were mainly positive (Figure S3a), while outside PA the trends were systematically lower (Figure S3c).


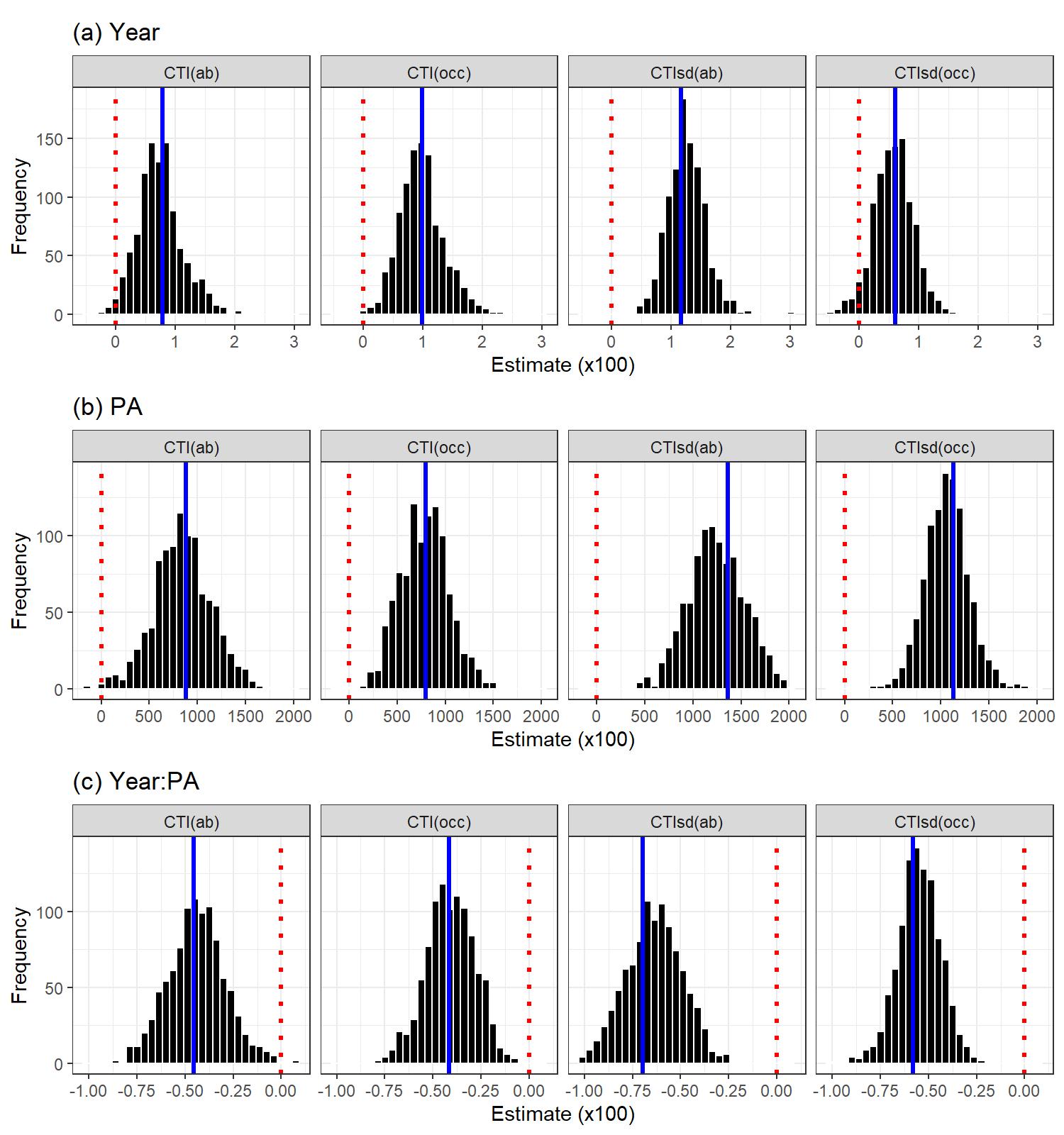
Figure S3: Histograms (in black) of the estimate distributions (multiplied by 100 to avoid decimals) for the CTI(occ), CTI(ab), CTI_sd_(occ) and CTI_sd_(ab) models investigating the (a) temporal trend, (b) the site protection status (PA) and (c) the interaction between site protection and year. Baseline is inside PA. The solid line (blue) represents the estimate of the model with all the species and the dotted line (red) represents the zero.

Devictor V, et al. 2012. Differences in the climatic debts of birds and butterflies at a continental scale. Nature climate change, 2(2), 121.

Godet, L., Jaffré, M., & Devictor, V. (2011). Waders in winter: long-term changes of migratory bird assemblages facing climate change. Biology letters, 7(5), 714-717.

Appendix 6: Protected area surfaces in the study area. Protected area surface (km²) of the studied sites is represented by points located at the centre of the corresponding cell (5°×5° resolution), which include both protected and not protected sites and at least 15 sites. The protected area surface corresponds to the sum of the studied PA surfaces per cell. The size of the points indicates the protected area surface size.


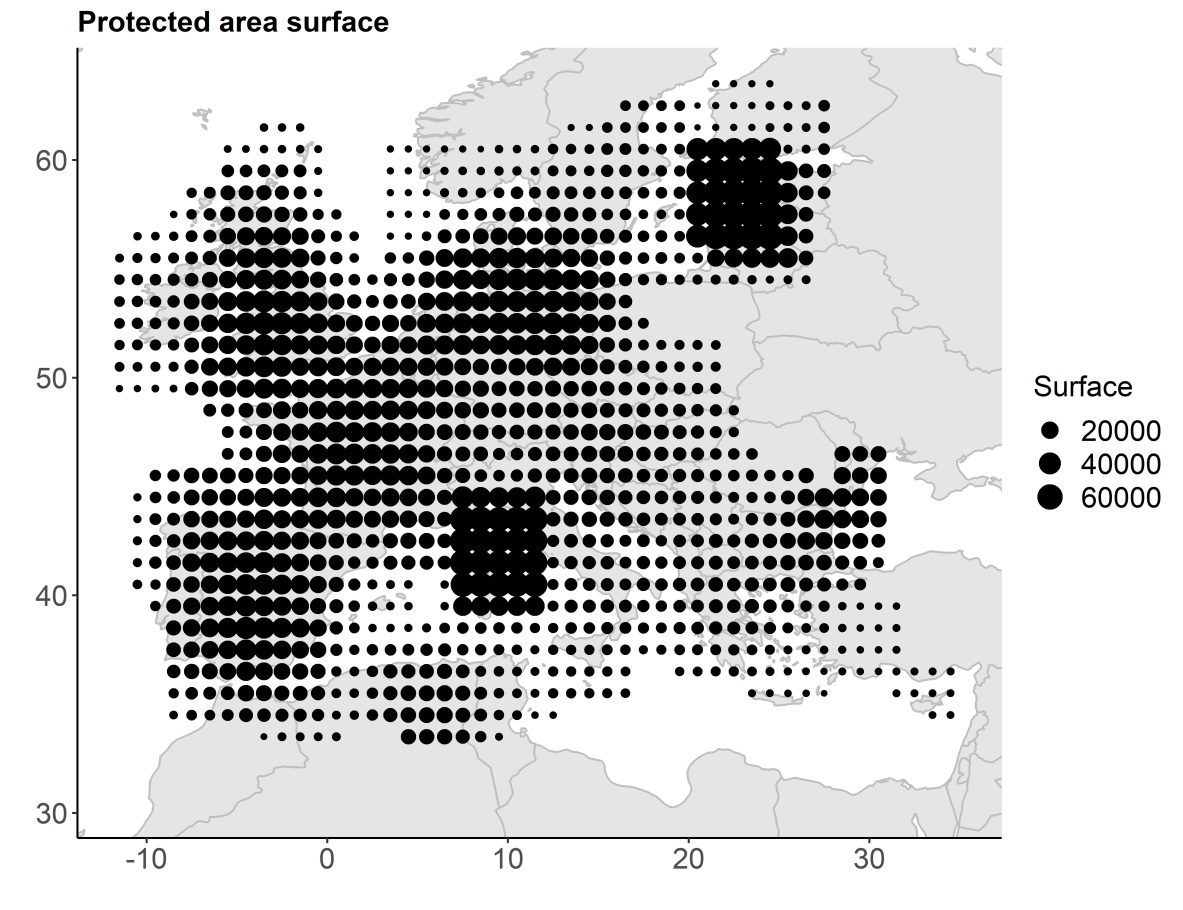

Supplement: Supplementary file 1 — Additional information is available online in the Supporting Information section at the end of the online article. The authors are solely responsible for the content and functionality of these materials. Queries (other than absence of the material) should be directed to the corresponding author. [file COBI-35-834-s001.docx]
